# Changes in immune checkpoint protein expression on CD8 T cells during neoadjuvant therapy in patients with pancreatic cancer

**DOI:** 10.1186/2051-1426-2-S3-P99

**Published:** 2014-11-06

**Authors:** Susan Tsai, Laura McOlash, Douglas B Evans, Bryon Johnson, Jill Gershan

**Affiliations:** 1 Medical College of Wisconsin, Milwaukee, WI, United States

## 

In most patients, pancreatic ductal adenocarcinoma (PDAC) is metastatic at diagnosis and available systemic therapies have had only a modest impact on survival duration. The overwhelming majority of patients with apparent localized, operable disease have extra-pancreatic micrometastases. Cancer patients experience both systemic and intra-tumor immune suppression. Thus, it is likely that pancreatic tumors evade immune surveillance. Harnessing the immune system to eradicate tumor cells represents a logical treatment approach and the optimal patient subset for study are those with operable disease. The immune profile of pancreatic cancer patients, especially those receiving active treatment, has not been studied. We are examining how neoadjuvant therapy affects the immunity of PDAC patients with low tumor burden and operable disease. We hypothesize that the changes in the peripheral immune profile that occur during neoadjuvant therapy will reflect host/tumor immunity, and that these changes will be associated with tumor response/relapse and patient survival. To address this hypothesis we are examining immune profiles in peripheral blood samples acquired before neoadjuvant therapy (baseline), after neoadjuvant therapy (but before surgery), and again after surgery. Our preliminary data shows that during neoadjuvant therapy, there is a reduction in the percentage of CD4 T cells and an increase in the percentage of CD8 T cells. In some patients the CD8 T cells up-regulate the PD-1 and 2B4 inhibitory receptors (referred to as immune checkpoint proteins). One interpretation of these findings is that neoadjuvant therapy facilitates immune activation. Patients that have increased expression of PD-1 and 2B4 on CD8 T cells may have more activated adaptive immunity induced by neoadjuvant therapy. Further patient follow-up will determine if this profile correlates with a more favorable disease outcome. Our preliminary data indicate that the neoadjuvant treatment period may be an opportune time to add immune therapy to release T cell immune suppression. We anticipate that our data will contribute to an understanding of how the immune system is altered during PDAC progression and will provide a rationale for the timing and delivery of novel anti-tumor immunotherapies to improve patient survival.

